# Estimating 10-Year Cardiovascular Disease Risk in Primary Prevention Using UK Electronic Health Records and a Hybrid Multitask BERT Model: Retrospective Cohort Study

**DOI:** 10.2196/76659

**Published:** 2025-11-13

**Authors:** Tianyi Liu, Lei Lu, Yanzhong Wang, Andrew J Krentz, Vasa Curcin

**Affiliations:** 1 Department of Population Health Sciences, School of Life Course & Population Sciences, King's College London, Addison House, Guy’s Campus, London, SE1 1UL, United Kingdom, 44 07422940311; 2 Metadvice, London, United Kingdom

**Keywords:** cardiovascular diseases, risk prediction, deep learning, electronic health records, health equity, survival analysis

## Abstract

**Background:**

Cardiovascular disease (CVD) remains a leading cause of preventable morbidity and mortality, highlighting the need for early risk stratification in primary prevention. Traditional Cox models assume proportional hazards and linear effects, limiting flexibility. While machine learning offers greater expressiveness, many models rely solely on structured data and overlook time-to-event (TTE) information. Integrating structured and textual representations may enhance prediction and support equitable assessment across clinical subgroups.

**Objective:**

This study aims to develop a hybrid multitask deep learning model (MT-BERT [multitask Bidirectional Encoder Representations from Transformers]) integrating structured and textual features from electronic health records (EHRs) to predict 10-year CVD risk, enhancing individualized stratification and supporting equitable assessment across diverse demographic groups.

**Methods:**

We used data from Clinical Practice Research Datalink (CPRD) Aurum comprising 469,496 patients aged 40‐85 years to develop MT-BERT for 10-year CVD risk prediction. Structured EHR variables and their corresponding textual representations were jointly encoded using a multilayer perceptron and a distilled version of the BERT model (DistilBERT), respectively. A fusion layer and stacked multihead attention modules enabled cross-modal interaction modeling. The model generated both binary classification outputs and TTE risk scores, optimized using a custom FocalCoxLoss function with uncertainty-based weighting. Prediction targets encompassed composite and individual CVD outcomes. Model performance was evaluated using the area under the receiver operating characteristic curve (AUROC), concordance index, and Brier score, with subgroup analyses by ethnicity and deprivation, and heterogeneity assessed using Higgins *I*² and Cochran Q statistics. Generalizability was assessed via external validation in a held-out London cohort.

**Results:**

The MT-BERT model yielded AUROC values of 0.744 (95% CI 0.738‐0.749) in males and 0.782 (95% CI 0.768‐0.796) in females on the test set (n=711,052), and 0.736 (95% CI 0.729‐0.741) and 0.775 (95% CI 0.768‐0.780), respectively in “spatial external” validation (n=144,370). Brier scores were 0.130 in males and 0.091 in females. Individuals classified as high-risk (≥40% risk in males and ≥34% in females) demonstrated significantly reduced 10-year event-free survival relative to lower-risk individuals (log-rank *P*<.001). Model performance was consistently higher in females across all metrics. Subgroup analyses revealed substantial heterogeneity across ethnicity and deprivation (*I*²>70%), especially among males, with lower AUROC in South Asian and Black ethnic groups. These findings reflect variation in model performance across demographic groups while supporting its applicability to large-scale CVD risk stratification.

**Conclusions:**

The proposed hybrid MT-BERT model predicts 10-year CVD risk for primary prevention by integrating structured variables and unstructured clinical text from EHRs. Its multitask design facilitates both individualized risk stratification and TTE estimation. While performance was modestly reduced in deprived and minority ethnic subgroups, these findings provide preliminary support for advancing equity-aware, data-driven prevention strategies in increasingly diverse health care settings.

## Introduction

### Background

Cardiovascular disease (CVD) is a major public health challenge, accounting for 1 in 4 deaths in the United Kingdom and affecting 7.6 million individuals. In 2022, over 1,00,000 hospital admissions were attributed to acute coronary syndromes [1]. Beyond morbidity and mortality, CVD imposes US $15 billion in annual health care costs and US $35 billion in economic losses. Additionally, 6‐8 million individuals have undiagnosed or uncontrolled hypertension, further increasing preventable CVD-related complications [1,2].

To address this, the United Kingdom has implemented national prevention strategies, with the UK’s National Institute for Health and Care Excellence (NICE) guidelines [3] recommending CVD risk assessments for individuals aged ≥40 years every 5 years via general practitioners (GPs). These assessments rely on structured clinical data and established 10-year risk prediction models such as QRISK3 [4] (developed and validated in UK populations) and Pooled Cohort Equations [5] (developed in US cohorts). Despite their widespread use, these conventional models rely on Cox regression, whose linear and proportional assumptions hinder their ability to model complex risk interactions, thereby limiting predictive performance in clinical practice [6,7].

Significant disparities in CVD burden persist. For instance, South Asians in the United Kingdom have nearly twice the coronary heart disease (CHD) incidence of White Europeans, with South Asian females experiencing rates similar to White European males [8]. Black African and Caribbean individuals do not show an elevated risk but receive fewer preventive treatments despite clinical indications [9]. Socioeconomic deprivation further exacerbates disparities, with higher CVD risk factor prevalence, hospital admissions, lower treatment uptake, and twice the mortality rate for individuals younger than 75 years in the most deprived areas [10]. Although conventional models can incorporate subgroup characteristics, they often lack the flexibility to model the complex, interdependent factors contributing to these disparities [11].

Machine learning (ML) approaches have emerged as promising alternatives to traditional CVD risk estimation models. A range of ML methods, from logistic regression and decision trees to ensemble techniques such as boosting, bagging, and stacking, have been explored for CVD risk prediction [6]. However, particularly in the context of primary prevention, many existing ML-based algorithms are limited by relatively small sample sizes, heterogeneous data quality, and a lack of consistent external validation, which may restrict their clinical applicability. Moreover, while some recent ML-based survival models, such as DeepSurv (Deep Neural Network–based Cox Model) [12], incorporate time-to-event (TTE) information, many CVD risk prediction algorithms continue to treat outcomes as binary events, without explicitly addressing censoring or time-dependent risk [7]. In addition, most models primarily use structured clinical variables and often overlook unstructured sources, such as physician notes and patient-reported symptoms, that could provide important contextual information for risk stratification [13].

Natural language processing (NLP), particularly Transformer-based models such as BERT [14] (Bidirectional Encoder Representations from Transformers), presents a novel approach to integrating unstructured clinical text into CVD risk prediction. Transformers use self-attention mechanisms to model long-range dependencies and complex relationships between variables. As an encoder-based Transformer model, BERT generates dense, context-rich embeddings by capturing bidirectional contextual information. Pretrained on large-scale corpora through transfer learning, BERT can effectively extract meaningful features from clinical text. Given that patient comorbidities, lifestyle factors, and social determinants are often recorded in unstructured formats, NLP models such as BERT can bridge critical gaps in cardiovascular risk assessment.

With increasing digital health adoption, NLP-driven models offer a scalable and cost-effective approach to CVD risk stratification. In particular, pretrained Transformer-based architectures such as BERT enable efficient fine-tuning on domain-specific datasets without the need for extensive retraining, reducing resource demands for clinical implementation. By integrating structured electronic health record (EHR) data with unstructured text sources such as patient interactions, chatbots, and clinical notes, future models could further enhance risk prediction and address limitations of conventional approaches. In this study, we emulate such integration by transforming structured variables into textual representations, providing an initial step toward leveraging both data modalities. Transformer-based models can better capture complex risk patterns across diverse populations, supporting more timely and equitable cardiovascular risk assessment for primary prevention.

### Rationale and Objectives

This study aims to develop a hybrid Multitask BERT model (MT-BERT) for prognostic CVD risk assessment, integrating structured and textual representations derived from UK EHR data using phenotyping techniques. The model is designed to support primary care decision-making by predicting 10-year CVD risk to guide early intervention, statin initiation, and lifestyle modification, potentially enhancing or replacing conventional statistical approaches.

While traditional survival models rely primarily on structured clinical variables, the proposed MT-BERT approach incorporates unstructured information (eg, physician notes and comorbidity narratives) to improve predictive performance, particularly among underrepresented high-risk populations.

To achieve this, we develop a model for both CVD outcome classification and TTE hazard ranking, enhancing individualized risk stratification and addressing biases in structured datasets. We also introduce a novel FocalCoxLoss function, enabling uncertainty-weighted joint optimization of classification and survival objectives. The study emphasizes model development, feature engineering, and optimization and includes external validation using data from a distinct London region to assess spatial generalizability. Additionally, we leverage longitudinal EHR data to better support practical implementation in primary care.

The specific objectives of this study were as follows:

1. Extract structured and unstructured predictors, CVD outcomes (binary classification and TTE data) from Clinical Practice Research Datalink (CPRD) Aurum [15] via phenotyping.
2. Develop the MT-BERT model integrating textual and structured variables for CVD risk prediction, incorporating the novel FocalCoxLoss for multitask learning.
3. Evaluate model performance across individual and composite CVD outcomes, with subgroup analyses by ethnicity and socioeconomic deprivation, focusing on discrimination, calibration, and fairness metrics.
4. Ensure transparent reporting according to the TRIPOD+AI (Transparent Reporting of a multivariable prediction model for Individual Prognosis Or Diagnosis + artificial intelligence) [16], including detailed documentation of feature engineering, data harmonization, and hyperparameter optimization.

The aim of this study was to evaluate the effectiveness of multimodal deep learning for long-term CVD risk prediction and to advance personalized health care through the integration of NLP and structured data from UK EHR.

## Methods

### Overview

This study follows the guidelines outlined in the TRIPOD+AI [16] statement (Checklist 1); an extension of the TRIPOD statement specifically designed for AI (including ML) prediction models.

### Study Design and Patient Records Inclusion

This study uses a retrospective EHR-based design, leveraging CPRD Aurum [15] (study registration protocol no.: 21_000346) to develop NLP-driven CVD risk prediction models using longitudinal patient records. CPRD Aurum is a routinely updated UK primary care database integrating deidentified patient records from 1359 GP practices (primary care sites), covering 16.6% of UK GPs and representing one-fifth of the UK population. It includes demographic data, diagnoses, symptoms, prescriptions, and dosages, standardized via MedCode ID to ensure interoperability with Read Code and SNOMED CT. As of January 2022 (version 2.7), it comprises over 40 million research-eligible patients with a median follow-up of 8.74 years (IQR 3.22-19.87).

The “External” (London) validation set represents a “spatial” external cohort from CPRD practices located in Greater London, selected to ensure demographic diversity, particularly higher proportions of ethnic minority patients.

**Figure 1.**
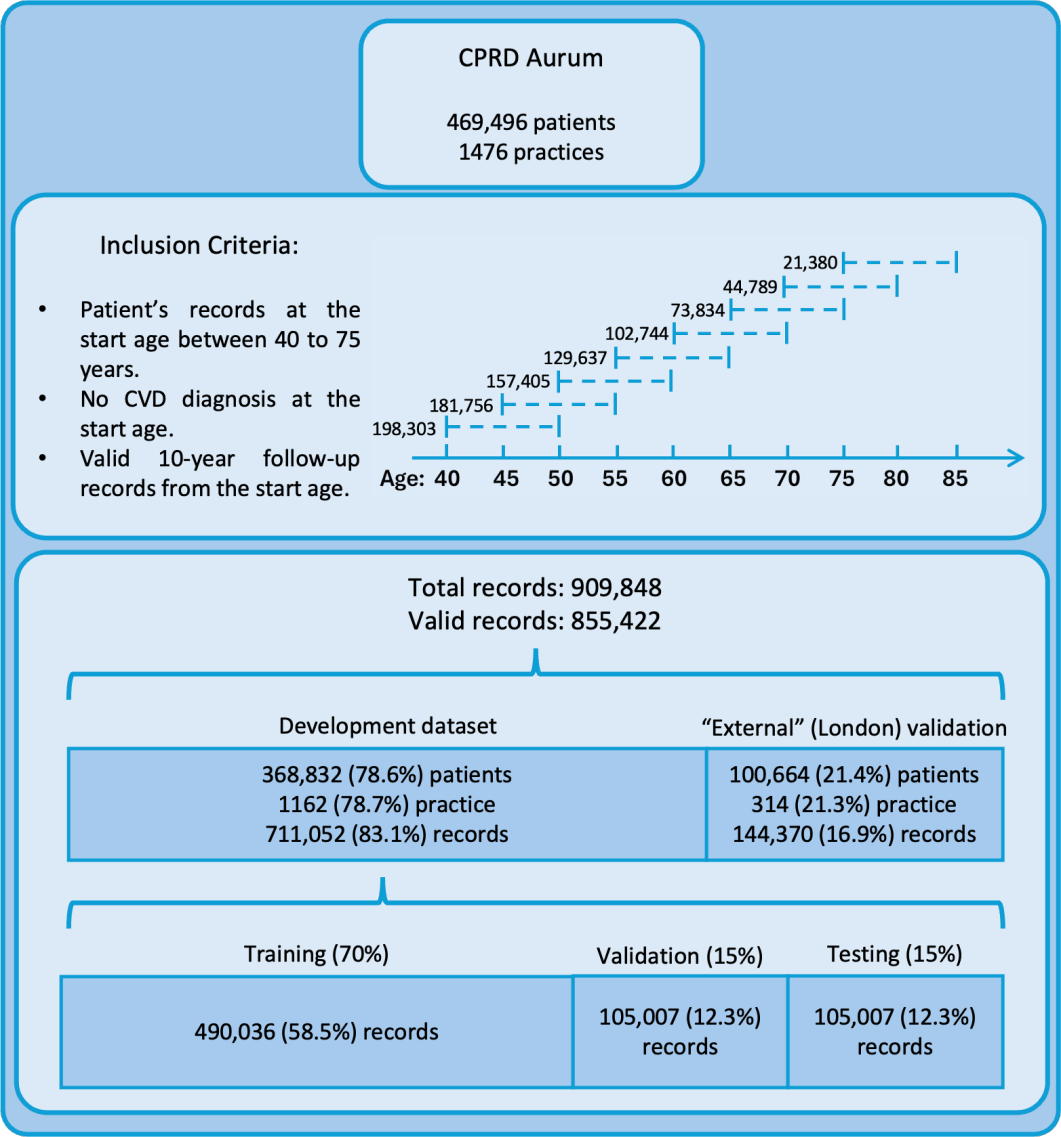
Workflow for study population and records extraction. CPRD: Clinical Practice Research Datalink; CVD: cardiovascular disease.

The data preprocessing workflow followed our prior work [17]. As shown in Figure 1, we selected a stratified random sample of 469,496 patients aged 40 to 85 years who were registered between 2011 and 2021. Unique CPRD identifiers were used to link records and reconstruct longitudinal health trajectories, ensuring a representative UK primary care cohort. Patients with at least one 10-year follow-up period were included to verify consistency in registration and clinical event timestamps. Follow-up intervals began at ages 40 to 75 years in 5-year increments, allowing individuals to contribute multiple independent 10-year records if they were CVD-free at entry. To emulate clinical practice, we extracted multiple fixed-horizon prediction episodes for each patient, reflecting periodic risk assessments recommended in the UK NICE guidelines [3] for adults aged 40 to 85 years. Consistent with our prior ML-based survival study, all baseline models were trained in exactly the same pipeline with identical predictors, censoring rules, preprocessing, and the same development and validation splits. This setup enables like-for-like comparisons and effectively represents structure-only, single-objective ablation settings against which MT-BERT is evaluated. While the guidelines do not specify data extraction procedures, this design strategy ensures alignment with real-world primary prevention workflows and enhances model relevance for general practice.

Given the scale and representativeness of CPRD Aurum, sample size adequacy was evaluated according to the framework proposed by Riley et al [17] for clinical prediction model development. The cohort substantially exceeded recommended thresholds for minimum events per predictor, enabling robust model development, rare event estimation, and stratified subgroup analyses.

### Extraction and Textual Transformation of Predictors

We extracted all predictor variables at the cohort entry year, primarily based on QRISK3 definitions, with minor adaptations where appropriate (Table 1). Phenotypes were defined using the HDRUK Phenotype Library [18], while comorbidities were identified through CArdiovascular disease research using LInked BEspoke studies and Electronic health Records (CALIBER, developed by the University College London Institute of Health Informatics [CALIBER research team]) [19], with corresponding *ICD-10* (*International Classification of Diseases, Tenth Revision*) codes detailed in Table S1 (Multimedia Appendix 1). MedCode IDs were documented and mapped to controlled terminologies to ensure consistency, facilitate updates, and enhance transparency (Multimedia Appendix 2).

Static variables (eg, gender, ethnicity, and deprivation) were assigned based on the most recent record available. Time-dependent variables (eg, smoking status and medication use) were determined within a 6-month window around the entry year; in the absence of recent records, smoking status was inferred from prior history, and medication use was classified based on any previous prescription. Comorbidities were considered present if recorded before cohort entry within the same time frame. Absence of records was interpreted as no occurrence rather than missing data.

For continuous variables (eg, BMI, systolic blood pressure, and total cholesterol/high-Ddensity lipoprotein ratio), if no values were available within the acceptable window, missing data were imputed using linear regression, provided that at least 3 prior measurements existed within a 2-year span.

To integrate established risk assessment into ML models, we derived an additional predictor based on the QRISK score calculated by an R package [20], represented in categorical form, aligned with UK clinical thresholds for statin treatment initiation.

Unlike traditional models based on structured data, this study transforms structured predictors into text representations for NLP-based modeling. To implement this approach, categorical variables including risk groups, ethnicity, smoking status, and comorbidities were converted into textual descriptions reflecting patient status at cohort entry, avoiding numerical encoding. Age and BMI were also expressed in textual form. Continuous variables were otherwise retained in their original form. The textual transformation template is provided in Table 1.

**Table 1.** Overview of predictors, outcome definitions, and preprocessing approaches.

Variable	Textual transformation/Preprocessing description
Continuous predictors	
Age	*“The patient is a {age_category}”*, where age < 45=young adult, 45‐59=middle-aged adult, 60‐74=young-old adult, and age ≥ 75=older adult
BMI (kg/m²)	Appended as: *“with a BMI classified as {bmi_category}”*, where BMI <18.5=underweight, 18.5‐24.9=normal weight, 25.0‐29.9=overweight (preobesity), 30.0‐34.9=Class 1 obesity, 35.0‐39.9=Class 2 obesity, BMI ≥40.0=Class 3 obesity (severe obesity)
Systolic blood pressure (mmHg)	Normalized to [0, 1] range using min-max scaling
SD of systolic blood pressure	Normalized to [0, 1] range using min-max scaling
Diastolic blood pressure (mmHg)	Normalized to [0, 1] range using min-max scaling
Total/High-density lipoprotein ratio	Normalized to [0, 1] range using min-max scaling
Townsend score	Normalized to [0, 1] range using min-max scaling
Categorical predictors	
Ethnicity (9 categories)	Appended as: *“and is of {ethnicity_group} ethnicity”*
Smoking status (5 categories)	Appended as: *“The patient is a {smoking_status}”*
Prestratified risk group (4 categories)	Appended as: *“Pre-stratified risk indicating a {risk_group}”*, where 0%‐4.9%%=low risk, 5.0%‐9.9%%=moderate risk, 10.0%‐19.9%%=high risk, and ≥20.0%=extreme high risk
Binary comorbidity predictors	
Type 1/2 diabetes mellitus	Appended as: *“The patient has a history of {condition_1, condition_2,.}”*
Chronic kidney disease stage 3, 4, or 5	Appended as: *“The patient has a history of {condition_1, condition_2, .}”*
Family history of coronary heart disease	Appended as: *“The patient has a history of {condition_1, condition_2, .}”*
Atrial fibrillation	Appended as: *“The patient has a history of {condition_1, condition_2, .}”*
Erectile dysfunction	Appended as: *“The patient has a history of {condition_1, condition_2, .}”*
HIV/AIDS	Appended as: *“The patient has a history of {condition_1, condition_2, .}”*
Migraine	Appended as: *“The patient has a history of {condition_1, condition_2, .}”*
Rheumatoid arthritis	Appended as: *“The patient has a history of {condition_1, condition_2, .}”*
Systemic lupus erythematosus	Appended as: *“The patient has a history of {condition_1, condition_2, .}”*
Severe mental illness	Appended as: *“The patient has a history of {condition_1, condition_2, .}”*
Antipsychotic	Appended as: *“The patient has a history of {condition_1, condition_2, .}”*
Corticosteroid	Appended as: *“The patient has a history of {condition_1, condition_2, .}”*
Treated hypertension	Appended as: *“The patient has a history of {condition_1, condition_2, .}”*
Cardiovascular disease outcomes	
Coronary heart disease	Used as binary outcome labels indicating disease presence (1=event occurred, 0=no event). Corresponding time-to-event (TTE) values were recorded in days, with a maximum follow-up of 3650 days for censored cases
Myocardial infarction	Used as binary outcome labels indicating disease presence (1=event occurred, 0=no event). Corresponding TTE values were recorded in days, with a maximum follow-up of 3650 days for censored cases
Ischemic stroke	Used as binary outcome labels indicating disease presence (1=event occurred, 0=no event). Corresponding TTE values were recorded in days, with a maximum follow-up of 3650 days for censored cases
Heart failure	Used as binary outcome labels indicating disease presence (1=event occurred, 0=no event). Corresponding TTE values were recorded in days, with a maximum follow-up of 3650 days for censored cases
Angina	Used as binary outcome labels indicating disease presence (1=event occurred, 0=no event). Corresponding TTE values were recorded in days, with a maximum follow-up of 3650 days for censored cases
Cardiovascular disease (QRISK)	A composite outcome including coronary heart disease (CHD), stroke, and transient ischemic attack (TIA). TTE was defined as the time to the earliest occurring event, in days
Cardiovascular disease (Composite)	A composite outcome including the above cardiovascular conditions, plus abdominal aortic aneurysm (AAA), and peripheral artery disease (PAD). TTE was defined as the time to the earliest occurring event, in days
Vascular dementia	Used as a binary outcome label indicating disease presence (1=event occurred, 0=no event). TTE was recorded in days, with a maximum follow-up of 3650 days for censored cases

### CVD Outcome and TTE Specification

Each CVD outcome (Table 1) was defined using phenotype-based criteria and assessed individually with its corresponding TTE, including CHD (*ICD-10*: I20-I25), stroke (I63-I66, I69), transient ischemic attack (G45-G46), heart failure (I50), abdominal aortic aneurysm (I71), and peripheral arterial disease (I73-I74). Additionally, 2 composite CVD outcomes, one aligned with QRISK and one containing all of the above, were defined based on the earliest occurrence of any included CVD event. Although not a classical CVD outcome, vascular dementia (F01) was included as an exploratory end point due to its close association with cerebrovascular disease. CVD events were recorded if they occurred within the 10-year follow-up or up to 6 months thereafter. Absence of records was interpreted as no event rather than missing data. The definition and mapping of CVD outcomes are detailed in Table S2 in Multimedia Appendix 1 and in Multimedia Appendix 2.

### Model Architecture and Training Strategy

**Figure 2.**
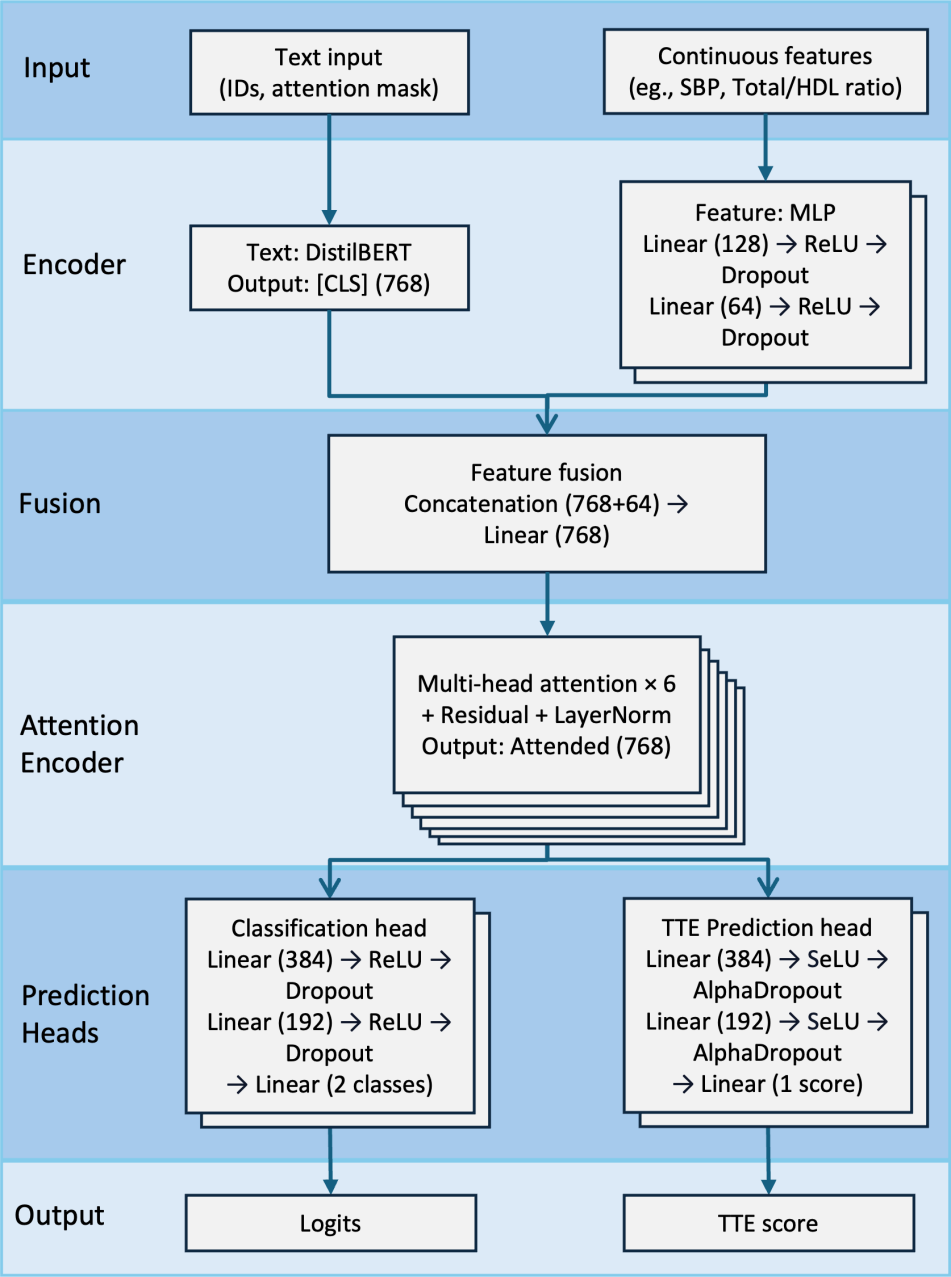
Architecture of the Multitask Bidirectional Encoder Representations from Transformers model for cardiovascular disease risk prediction. HDL: high-density lipoprotein; MLP: multilayer perceptron; SBP: systolic blood pressure; TTE: time-to-event.

As shown in Figure 2, we developed an MT-BERT framework for the prediction of CVD outcomes, which integrates a distilled version of the BERT model (DistilBERT) [21] for textual features and a multilayer perceptron (MLP) for structured clinical predictors, incorporating multihead attention and residual connections to refine feature interactions. DistilBERT retains representational power while reducing computational complexity by removing token-type embeddings and the pooler layer. Unlike conventional concatenation-based approaches, our model applies a transformation layer for feature alignment before passing the fused representation through stacked attention layers, enabling dynamic cross-modal refinement. The MLP module, implemented in PyTorch, consists of fully connected layers with Rectified Linear Unit activations and dropout, ensuring structured feature regularization. For prediction, the model simultaneously outputs binary classification logits (CVD presence) and a log hazard score (TTE) for survival analysis, with temperature scaling improving classification stability. Full implementation details are provided in Table S3 (Multimedia Appendix 1).

Structured features were MinMax scaled, and text data were tokenized using the DistilBERT tokenizer. The dataset was split in a 7:2:1 ratio into training, validation, and testing sets (Figure 1) using stratified sampling to maintain class balance. Labels and TTE values were converted into tensors for model compatibility. Class imbalance was addressed with a dynamically adjusted weighting scheme, scaling standard balanced class weights based on the observed event rate. The processed data were structured into TensorDatasets and loaded for training.

Our model uses a customized FocalCoxLoss, a multitask loss function that combines Focal Loss, a class imbalance-aware variant of cross-entropy loss, for event classification, which optimizes decision boundaries, and Cox Loss, as used in DeepSurv [12], for survival analysis, which optimizes risk ranking. The 2 components are balanced using learnable uncertainty-based weighting [22] as follows:

$L\;=\;\left(\frac{1}{2\sigma_{focal}^{2}}\right)L_{focal}+\;\left(\frac{1}{2\sigma_{cox}^{2}}\right)L_{cox}+\log\sigma_{focal}+\log\sigma_{cox}$

where $\sigma_{focal}$ and $\sigma_{cox}$ are learnable task-specific variance parameters. Unlike fixed-weight loss functions, this approach enables dynamic adjustment of task contributions by assigning higher effective weights to tasks with lower predicted uncertainty, thereby optimizing the trade-off between binary CVD event prediction and TTE risk estimation during training.

To ensure stability, we adopted a 2-stage training strategy. Initially, was frozen, maintaining a fixed loss ratio to prevent early instability. After 10 epochs, it was unfrozen, allowing adaptive loss scaling. DistilBERT remained trainable throughout, enabling concurrent optimization of textual and structured representations.

Training used AdamW with cosine annealing, gradient clipping, and early stopping for stable convergence. Dropout rates were optimized via Optuna hyperparameter tuning, while learning rate (initially 3e-4) and weight decay (1e-4) were manually set. During training, the learning rate was dynamically adjusted using a cosine annealing schedule with warm restarts. Model selection was based on the area under the curve and C-statistic maximization.

### Performance Evaluation, Generalizability, and Fairness

Model performance was assessed on the held-out test set using the optimal hyperparameters identified during training (Figure 1). Calibration was performed via isotonic regression and assessed using Brier scores and calibration plots across risk deciles. Discrimination was quantified by the AUROC and the C-statistic, with 95% CIs estimated via 1000 bootstrap resamples. Internal validity was assessed through 10-fold cross-validation. Classification metrics, including accuracy, recall, and specificity, were reported at the optimized threshold. Kaplan-Meier (KM) survival curves stratified by predicted risk groups were used to evaluate risk separation and clinical utility.

To evaluate generalizability and fairness, we performed “spatial external” validation using a London held-out cohort (Figure 1), selected for its higher proportion of ethnic minority patients. AUROC with 95% CIs was computed across all 9 ethnicity categories and Townsend deprivation deciles. In parallel, we reported aggregated subgroup results for broader ethnicity groupings including White, South Asian (Indian, Pakistani, and Bangladeshi), and Black (African and Caribbean). We further evaluated performance variation across individual Townsend quintiles. To quantify heterogeneity in model performance, Higgins *I*² statistic and Cochran Q values [23] were computed across ethnicity and deprivation groups, where the Higgins *I*² statistic quantifies the percentage of total variation across studies due to heterogeneity rather than chance, and the Q value tests whether the observed differences in study results are due to chance or true heterogeneity.

### Ethical Considerations

This study uses CPRD Aurum data under protocol number 21_000346, which was reviewed and approved by the Independent Scientific Advisory Committee (ISAC) on behalf of the Medicines and Healthcare Products Regulatory Agency. The study relies on anonymized patient records and follows ethical guidelines for research using deidentified health care data. No patient or public consent was required, as the research was conducted using fully anonymized data. There was no direct patient or public involvement in the study’s design, execution, analysis, or dissemination. No financial compensation was provided.

## Results

### Baseline Characteristics of Study Population

We extracted 909,848 records from 469,496 patients across 1476 practices in England (Figure 1). After quality control to remove records with extreme values for continuous features, 855,422 valid records were retained. Of these, 144,370 records (16.9%) from practices located in London were held out as the spatial external validation set. The remaining 711,052 records (83.1%) constituted the development dataset, which was randomly split into training, validation, and testing sets using a 70:15:15 ratio.

Table 2 summarizes the background characteristics of both the development dataset and the spatial validation set, disaggregated by gender. The statistics show that most comorbidities and CVD outcomes are significantly more common among males, which makes gender-specific modeling indispensable for this task. In addition, a substantially higher proportion, approximately one-third of individuals from minority ethnic backgrounds, is observed in the spatial validation set, whereas over 90% of the development dataset is White. This demographic contrast supports the use of the London practice cohort as a spatially distinct external validation set, particularly for assessing model generalizability and fairness in managing CVD risk among ethnic minority populations.

**Table 2.** Baseline characteristics of the study population by gender.

Characteristics	Male (n=403,545, 47.17%)		Female (n=451,877, 52.82%)	
	Development (n=335,891)	Spatial validation (n=67,654)	Development (n=375,161)	Spatial validation (n=76,716)
Continuous features, mean (SD)				
Age	50.62 (9.16)	50.51 (9.10)	51.29 (9.51)	51.13 (9.43)
BMI	27.45 (4.42)	27.25 (4.31)	27.18 (5.47)	26.54 (5.30)
Systolic blood pressure	135.17 (12.30)	134.79 (12.02)	131.32 (14.83)	129.63 (14.88)
SD of systolic blood pressure	9.05 (4.83)	8.93 (4.88)	9.87 (4.88)	9.50 (4.79)
Diastolic blood pressure	81.82 (7.42)	81.75 (7.33)	79.18 (7.93)	78.47 (7.93)
Total/High-density lipoprotein ratio	4.08 (0.83)	4.20 (0.84)	4.11 (0.84)	4.19 (0.85)
Categorical features, %				
Ethnicity				
White	93.44	64.82	93.92	65.65
Indian	1.50	8.33	1.46	8.32
Pakistani	1.02	2.05	0.91	1.62
Bangladeshi	0.26	1.35	0.22	1.18
Chinese	0.23	0.96	0.28	1.04
Other Asian	0.50	4.40	0.47	4.28
Black Caribbean	0.50	5.32	0.58	6.57
Black African	0.35	6.73	0.30	6.10
Other ethnic group	2.20	6.04	1.84	5.24
Smoking status				
Nonsmoker	45.73	47.10	58.42	59.71
Ex-smoker	51.73	50.71	39.40	38.50
Light smoker	0.61	0.47	0.75	0.63
Moderate smoker	1.17	1.04	0.99	0.80
Heavy smoker	0.76	0.67	0.44	0.35
Risk group				
Low risk	41.97	44.54	57.11	61.42
Moderate risk	26.67	26.74	22.59	21.51
High risk	21.63	20.53	14.98	13.07
Extreme high risk	9.73	8.18	5.32	3.99
Binary comorbidity, %				
Type 1 and 2 diabetes mellitus	4.75	3.82	3.61	2.82
Chronic kidney disease stage 3, 4, or 5	0.89	0.89	1.31	1.52
Family history of coronary heart disease	3.72	2.72	4.80	3.23
Atrial fibrillation	0.80	0.92	0.45	0.46
Erectile dysfunction	4.12	4.25	NA	NA
HIV/AIDS	0.12	0.05	0.03	0.03
Migraine	2.36	2.91	6.40	7.17
Rheumatoid arthritis	0.37	0.35	0.89	0.79
Systemic lupus erythematosus	0.05	0.05	0.21	0.22
Severe mental illness	0.96	0.68	0.96	0.84
Antipsychotic	0.42	0.34	0.42	0.37
Corticosteroid	3.22	2.53	4.68	3.87
Treated hypertension	13.83	12.72	14.43	13.01
Cardiovascular disease outcomes, %				
Cardiovascular disease (QRISK)	8.19	6.84	4.91	3.57
Cardiovascular disease (Composite)	10.87	9.04	6.59	4.97
Coronary heart disease	4.80	3.65	2.47	1.52
Myocardial infarction	2.60	2.13	0.98	0.76
Stroke	2.26	1.97	1.73	1.34
Transient ischemic attack	1.38	1.31	1.14	0.92
Abdominal aortic aneurysm	0.46	0.37	0.08	0.09
Peripheral artery disease	1.27	0.85	0.62	0.49
Heart failure	1.28	1.02	0.97	0.65
Angina	3.78	3.37	2.26	1.61
Vascular dementia	0.75	0.63	0.91	0.77

### Model Predictive Performance and Risk Stratification

We developed the MT-BERT model for multiple CVD outcomes, and all performance metrics were derived from the held-out test set unless otherwise stated (Table 3), including discrimination, calibration, and classification results for both composite and individual outcomes.

For both genders, the model trained to predict the composite CVD outcome achieved the highest discrimination. In males, the AUROC was 0.744 (95% CI 0.738‐0.749), and in females, 0.782 (95% CI 0.768‐0.796), higher than models trained on QRISK-defined CVD outcomes (males: 0.723, 95% CI 0.717‐0.732; females: 0.767, 95% CI 0.760‐0.777). The corresponding concordance index (C-index) values were 0.713 (95% CI 0.710‐0.716) in males and 0.732 (95% CI 0.725‐0.742) in females for composite CVD.

Among individual outcomes, ischemic stroke (males: 0.738, females: 0.769) and CHD (males: 0.732, females: 0.769) demonstrated relatively strong discriminatory performance. Angina also showed acceptable discrimination (males: 0.713, females: 0.741). In contrast, myocardial infarction exhibited lower discrimination (males: 0.679, females: 0.717), particularly in males, where recall was high but overall accuracy was reduced. Vascular dementia was additionally explored on an experimental basis and showed overall limited performance across evaluation metrics, similar to the pattern observed for myocardial infarction.

Continuous variables are presented as mean values with 2 decimal places for consistency. Individual clinical measurements (eg, blood pressure) are typically recorded as integers, but decimal values arise when reporting cohort-level averages.

“Cardiovascular disease (QRISK)” is a composite outcome including CHD, stroke, and transient ischemic attack. “Cardiovascular disease (Composite)” includes the above conditions plus abdominal aortic aneurysm and peripheral artery disease. Benchmark model results were adapted from our prior work [24], where models were trained and evaluated using the same CPRD Aurum cohort and standardized preprocessing for consistent comparability. RSF (Random Survival Forest), GBSA (Gradient Boosted Survival Analysis), XGBS (Extreme Gradient Boosted Survival Model), DeepSurv, and DeepHit (Deep Neural Network for Competing Risks).

KM curves are plotted with a full 0‐1 *y*-axis; shaded bands denote 95% CIs. Insets provide a zoomed view of the upper survival range for readability. Segments after time points with <10 individuals at risk are suppressed. High- versus low-risk groups use sex-specific thresholds (men≥40%, women≥34%). *P* values are from log-rank tests.

**Table 3.** Performance metrics of the Multi-Task Bidirectional Encoder Representations from Transformers model across cardiovascular disease outcomes by gender (test set).

Predicted outcome	AUROC^a^ (95% CI)	C-index^b^ (95% CI)	Brier score	Accuracy	Specificity	Recall	Baseline threshold (%)
Male							
CVD^c^ (Composite)	0.744 (0.738- 0.749)	0.713 (0.710- 0.716)	0.130	0.823	0.874	0.405	0.40
CVD (QRISK)	0.723 (0.717- 0.732)	0.699 (0.696- 0.702)	0.119	0.733	0.750	0.548	0.34
CHD^d^	0.732 (0.726- 0.739)	0.707 (0.704- 0.709)	0.107	0.782	0.795	0.518	0.34
Stroke	0.738 (0.702- 0.764)	0.688 (0.684- 0.692)	0.065	0.676	0.676	0.656	0.33
MI^e^	0.679 (0.670- 0.694)	0.667 (0.663- 0.674)	0.133	0.125	0.102	0.958	0.35
Angina	0.713 (0.702- 0.723)	0.701 (0.695- 0.706)	0.104	0.765	0.775	0.517	0.33
Female							
CVD (Composite)	0.782 (0.768- 0.796)	0.732 (0.725- 0.742)	0.091	0.861	0.887	0.487	0.34
CVD (QRISK)	0.767 (0.760- 0.777)	0.752 (0.749- 0.757)	0.077	0.914	0.9456	0.2950	0.31
CHD	0.769 (0.750- 0.785)	0.732 (0.725- 0.742)	0.059	0.872	0.883	0.427	0.28
Stroke	0.769 (0.717- 0.852)	0.737 (0.732- 0.745)	0.018	0.934	0.936	0.256	0.23
MI	0.717 (0.680- 0.761)	0.687 (0.682- 0.696)	0.078	0.408	0.403	0.900	0.27
Angina	0.741 (0.731- 0.754)	0.721 (0.718- 0.724)	0.070	0.878	0.887	0.387	0.29

a AUROC: area under the receiver operating characteristic curve.

b C-index: concordance index.

c Cardiovascular disease (QRISK)” is a composite outcome including coronary heart disease (CHD), stroke, and transient ischemic attack (TIA). “Cardiovascular disease (Composite)” includes the above conditions plus abdominal aortic aneurysm (AAA) and peripheral artery disease (PAD).

d CHD: coronary heart disease.

e MI: myocardial infarction.

**Figure 3.**
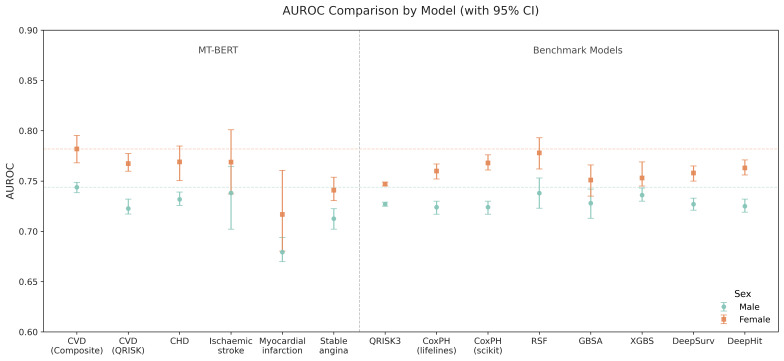
Discrimination performance of the Multitask Bidirectional Encoder Representations from Transformers model across cardiovascular disease outcomes, in comparison with benchmark conventional and machine learning models. AUROC: area under the receiver operating characteristic curve; CHD: coronary heart disease; CVD: cardiovascular disease; DeepSurv: Deep Neural Network–based Cox Model; DeepHit: Deep Neural Network for Competing Risks; GBSA: Gradient Boosted Survival Analysis; RSF: Random Survival Forest; XGBS: Extreme Gradient Boosted Survival Model.

The left panel of Figure 3 visualizes the AUROC values with 95% CIs across all CVD outcomes by gender, aligning with the summary in Table 3. The model showed the clearest advantage for composite CVD, with a higher AUROC than both QRISK-defined and individual outcome-specific models.

Across all outcomes, C-index values were consistently 0.01‐0.05 lower than AUROC. Model performance was generally higher in females across AUROC, C-index, and accuracy. The thresholds used to classify positive cases ranged from 0.23% to 0.40% in males and 0.23% to 0.34% in females, with higher thresholds observed in males, consistent with their event distribution and calibration characteristics.

In the right panel of Figure 3, the MT-BERT model is compared against benchmark models, including conventional approaches (QRISK3 [4] and CoxPH [25]), ensemble-based methods (RSF, GBSA, and XGBS), and deep learning models (DeepSurv [12] and DeepHit [26]). These benchmark models were implemented based on our previous work [24]. In this evaluation, the hybrid model consistently outperformed all comparators, with the greatest AUROC gains observed for composite CVD, especially in females.

**Figure 4.**
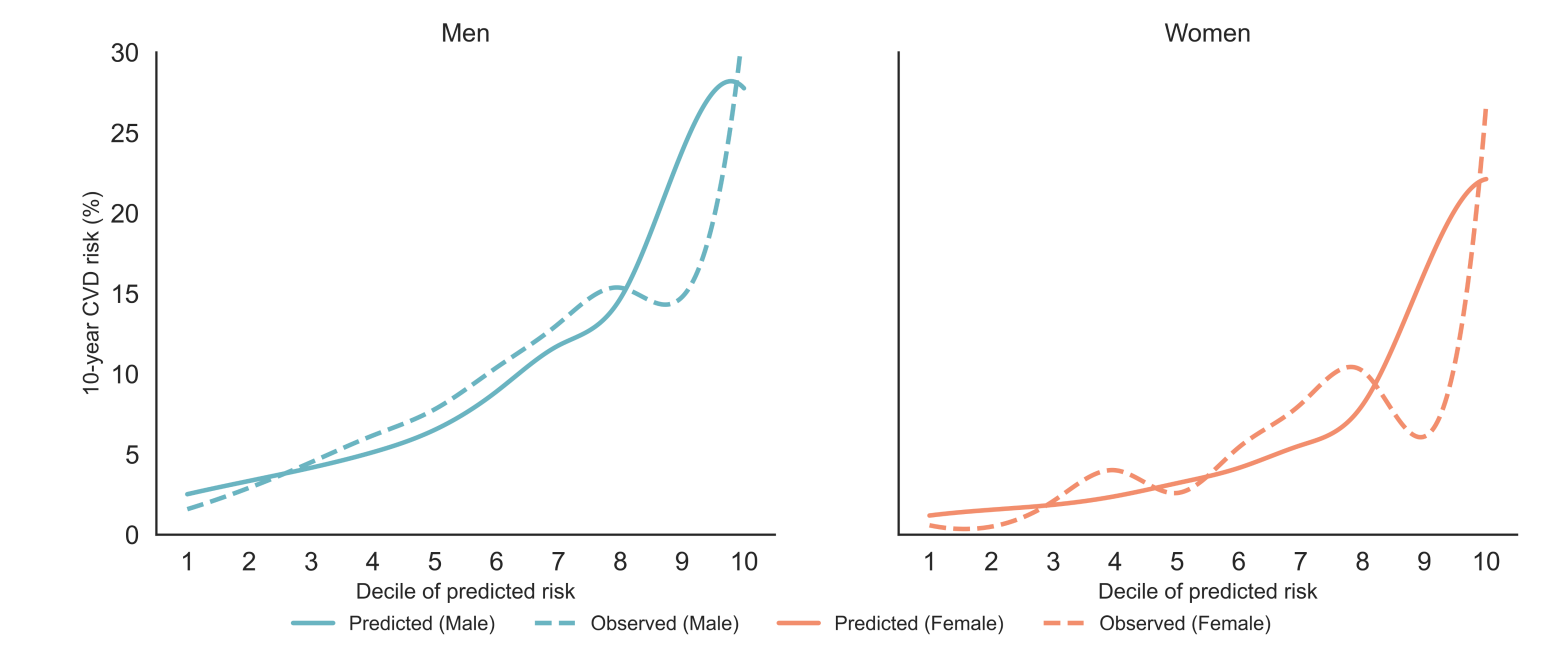
Calibration plots for 10-year cardiovascular disease risk by gender. CVD: cardiovascular disease.

Figure 4 presents the calibration plot for the predicted 10-year composite CVD risk using the MT-BERT model, stratified by gender. Predicted risks and observed event rates were plotted across deciles of predicted risk. In both genders, predicted risks increased monotonically across deciles, indicating good overall calibration. However, risk overestimation was evident in the upper deciles, particularly among males, suggesting inflation at the highest predicted risk levels. In females, predicted and observed risks were closely aligned in lower-risk and midrisk ranges, though moderate overprediction was also observed at the top deciles, where event rates were more variable. In particular, a dip in the observed rates was seen in the ninth decile, which we attribute to event sparsity and statistical variability in the highest risk groups. These findings are consistent with the Brier scores in Table 3, where males had a score of 0.130 and females had 0.091, reflecting slightly better overall calibration in women. Taken together, the hybrid model provided reasonably calibrated risk estimates for composite CVD, with modest overprediction at higher predicted risk levels, especially in males.

**Figure 5.**
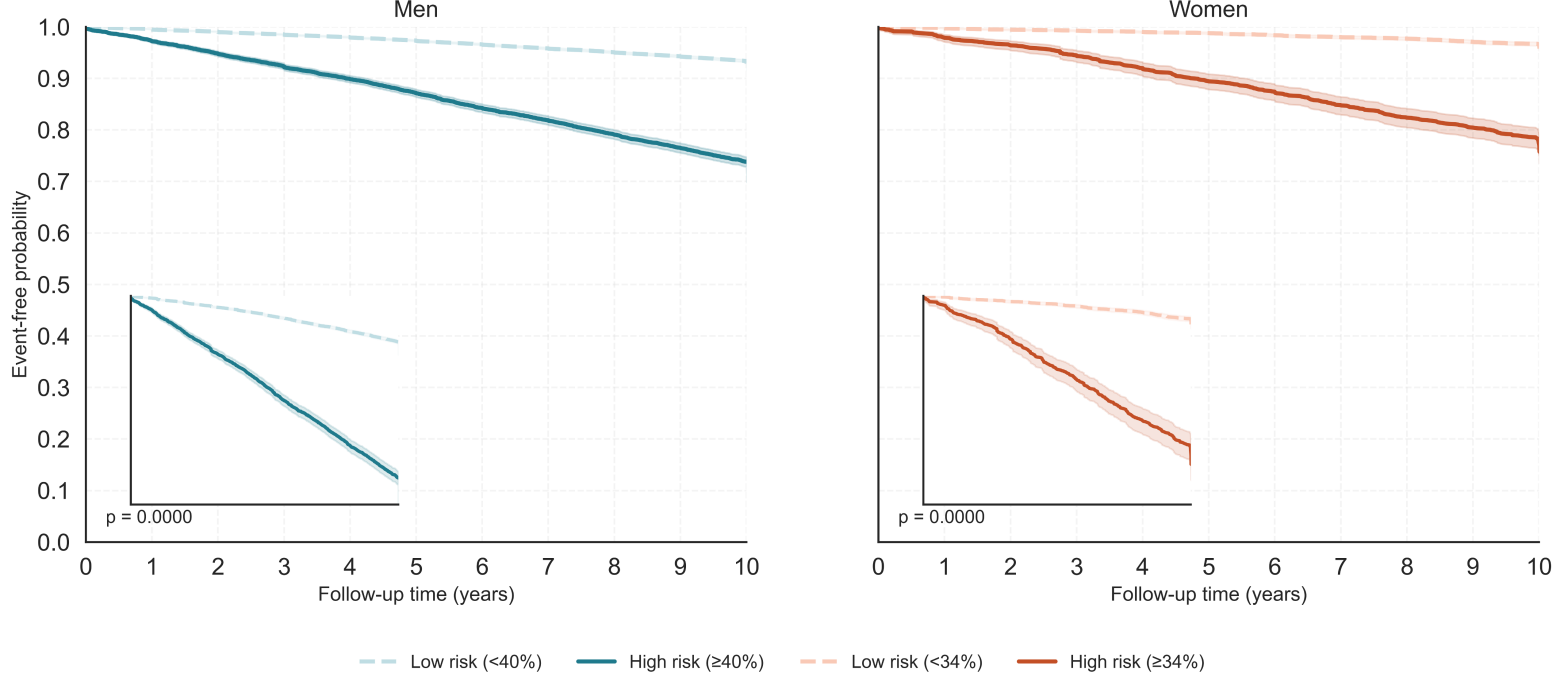
Kaplan-Meier survival curves for 10-year cardiovascular disease risk, stratified by predicted risk groups by gender.

KM survival curves for 10-year composite CVD risks are presented in Figure 5, stratified by predicted risk groups in both genders. Participants were classified into high- and low-risk categories using gender-specific thresholds: 40% or greater for men and 34% or greater for women. In both genders, individuals in the high-risk group exhibited significantly lower event-free survival over time, confirming the model’s ability to stratify patients according to long-term risk. Among males, the 10-year event-free probability declined to approximately 74% in the high-risk group, compared to over 93% in the low-risk group. A similar pattern was observed in females, with high-risk individuals showing notably steeper declines in survival probability. Log-rank tests yielded *P* values <.001 in both genders, indicating statistically significant separation between risk groups. These results support the prognostic utility of the model’s predicted risk scores, demonstrating effective stratification for composite CVD outcomes over a 10-year follow-up period.

Ethnicity (n=9) subgroups include White, Indian, Pakistani, Bangladeshi, Chinese, Other Asian, Black Caribbean, Black African, and Other ethnic groups. Ethnicity (n=3) aggregates broader categories into White, Asian (including Indian, Pakistani, Bangladeshi, Chinese, and Other Asian), and Black (Caribbean and African). Deprivation (n=5) refers to Townsend quintiles, while deprivation (n=10) refers to Townsend deciles.

### Model Fairness and Generalizability Across Demographics

**Figure 6.**
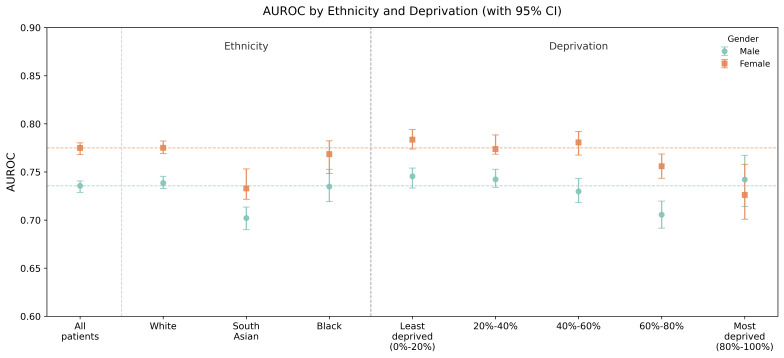
Assessment of model fairness across ethnic and deprivation subgroups (“spatial” validation set). AUROC: area under the receiver operating characteristic curve.

We assessed the performance of the MT-BERT model on the composite CVD outcome across ethnic and deprivation subgroups in the spatial validation set, separately by gender. Figure 6 shows AUROC values with 95% CIs for major ethnic and deprivation categories. Table 4 presents *I*² and Q statistics based on both broad and more granular subgroup definitions. All results pertain to the composite CVD outcome.

Among all patients, the AUROC for composite CVD was 0.736 (95% CI 0.729‐0.741) in males and 0.775 (95% CI 0.768‐0.780) in females. These represent a moderate decline compared to the internal test set, though overall generalizability remained acceptable. Other performance metrics are provided in Table S4 in Multimedia Appendix 1, where similar trends were observed—slight reductions compared to internal validation, but performance remained within a reasonable range. Across all subgroups, model discrimination was consistently higher in females than in males (Figure 6), with an average AUROC difference of approximately 0.04‐0.05 between genders. This pattern was consistent with results from the training, validation, and test sets (Table S4 in Multimedia Appendix 1; Table 3).

In males, model performance in the White ethnic group was marginally higher than in the overall male cohort, though the difference was not statistically significant. AUROC in the Black ethnic group was slightly lower, but the CIs overlapped with the overall population, indicating broadly comparable performance. The lowest AUROC was observed in the South Asian group, where a more pronounced decline was seen, and the CIs did not overlap with those of the White or overall male population, suggesting reduced discriminative ability in this subgroup. In females, similar patterns were observed. AUROC remained relatively consistent across the 3 main ethnic groups, though a modest decline was again noted in South Asian females, and a more notable drop was observed in Black females. When aggregated into 3 ethnic groups, *I*² values were 93.03% in males and 91.41% in females, indicating substantial heterogeneity (Table 4). Even with finer stratification into 9 ethnic groups, *I*² remained elevated (71.51% in males and 77.06% in females), suggesting that differences in model discrimination were not solely driven by broad grouping.

For deprivation, Figure 6 shows a decreasing trend in AUROC from the least to the most deprived groups. In females, AUROC declined steadily across the most deprived 40%. In males, performance dropped notably in the 60%‐80% deprivation quantile but showed partial recovery in the most deprived group. Correspondingly, heterogeneity in deprivation-based analyses remained high, with *I*² values of 83.34% (quintile-based) and 74.91% (decile-based) in males, and 82.73% and 81.55%, respectively, in females (Table 4).

Overall, heterogeneity across both ethnicity and deprivation subgroups was notable, with *I*² consistently above 70% and all Q-tests statistically significant (*P*<.001), except for the 9-category ethnicity analysis in males. These findings suggest that while the model maintained acceptable generalizability, subgroup-level variability in performance exists and may warrant further investigation in future work.

**Table 4.** Heterogeneity (*I*² and Q Statistics) by ethnicity and deprivation subgroups in the “spatial” validation set.

Subgroup (n)	Male		Female	
	*I*² (%)	Q (*P* value)	*I*² (%)	Q (*P* value)
Ethnicity (3)	93.03	28.70 (*P*<.001)	91.41	23.28 (*P*<.001)
Ethnicity (9)	71.51	28.08 (*P*<.001)	77.06	34.87 (*P*<.001)
Deprivation (5)	83.34	24.01 (*P*<.001)	82.73	23.16 (*P*<.001)
Deprivation (10)	74.91	35.87 (*P*<.001)	81.55	48.78 (*P*<.001)

## Discussion

### Principal Findings

The proposed hybrid MT-BERT model demonstrated strong performance in predicting 10-year CVD risk by integrating structured variables and free-text clinical notes from EHRs. While previous studies have explored the use of clinical text for risk prediction [27,28], few have focused on long-term CVD outcomes or used integrated multitask modeling. In this study, the model achieved high discrimination, demonstrated good calibration across predicted risk groups, and enabled effective survival stratification based on KM curves. Compared to conventional and ML-based survival models, the inclusion of unstructured text improved performance, particularly in females. These findings were consistent across both the internal test set and a demographically distinct “spatial external” validation cohort from London, supporting the model’s generalizability and potential clinical utility.

A key methodological contribution of this study is the integration of structured and unstructured EHR data within a BERT-MLP architecture, optimized through a unified multitask learning framework. Compared to categorical or binary representations, textual input can provide richer information about disease severity, temporal context, or interactions between conditions that structured variables may not explicitly encode. Moreover, transforming structured features into natural language text reduces data sparsity and produces denser, more learnable representations [29]. This transformation is particularly suited for BERT, which is optimized for NLP and benefits from pretrained language representations that enhance its ability to model semantic relationships, even in low-resource settings or when encountering rare comorbidities [30]. Thus, our approach allows for both increased model expressiveness and improved generalization to underrepresented patient profiles.

Building on prior work in deep survival modeling, such as DeepSurv [12] and DeepHit [26], the model incorporates a classification objective to support threshold-based clinical decision-making. By jointly minimizing a combined Focal and Cox-based loss, it enables simultaneous optimization of binary classification (AUROC) and survival risk ranking (C-index). This dual-objective design allows the model to capture both event occurrence and timing, enhancing its applicability in real-world risk stratification.

To ensure comparability, data extraction followed the protocol from our previous work [24] on ML-based survival models for CVD, maintaining consistency in preprocessing and enabling direct performance comparisons. Under this aligned experimental setup, the hybrid model outperformed earlier conventional and ML-based models, demonstrating the effectiveness of combining representation learning with multiobjective optimization. Importantly, these comparators already realize the principal ablation settings within an identical pipeline, namely, structure-only survival modeling with a single Cox objective (CoxPH, RSF, and DeepSurv). They therefore provide a pragmatic assessment of modality and loss without reimplementing stripped-down variants of MT-BERT.

Furthermore, compared to training separate models for classification and survival analysis, our multitask learning framework offers several advantages. First, by sharing representations between tasks, the model captures both static risk factors and temporal dynamics more efficiently, leveraging complementary information to enhance overall predictive performance [31,32]. Binary classification and survival modeling address distinct but related goals [12]: classification focuses on identifying individuals at high immediate risk, while survival modeling captures the time-dependent risk trajectory. Learning these jointly allows the model to optimize decision thresholds and risk ranking simultaneously. Additionally, shared representations act as a form of regularization, improving generalization especially in underrepresented subgroups [31]. Although we did not run a full factorial ablation, the existing comparators already realize the principal ablation settings within the same pipeline, namely, structure-only survival modeling with a single Cox objective. The consistent gains in both discrimination and calibration indicate a benefit from multitask integration, and we will conduct targeted ablations in future work to quantify incremental contributions beyond these baselines.

Despite these overall gains, subgroup analyses revealed reduced discrimination in certain populations, particularly males from deprived and ethnic minority groups. Similar disparities have been observed in other clinical ML applications [33], underscoring the importance of fairness-aware evaluation and algorithm refinement. Substantial heterogeneity (*I*²>70%) across ethnicity and deprivation strata further highlights the need for subgroup-specific performance auditing. These findings emphasize the necessity of addressing demographic variability to support equitable deployment of predictive models in diverse populations.

### Limitations

This study has several limitations that merit consideration. While the hybrid MT-BERT architecture offers enhanced representational capacity by integrating free-text and structured data, it also introduces considerable complexity [13,29]. BERT-based models are computationally intensive, requiring substantially more memory and processing time than MLPs trained solely on structured inputs. The need to process long token sequences increases both training and inference latency, which may limit scalability in resource-constrained environments. Additionally, textual representations derived from BERT may suffer from noise due to variability in clinical phrasing and tokenization. For instance, expressions such as “No history of diabetes” and “Diabetes: none” may yield inconsistent embeddings, introducing ambiguity in comorbidity interpretation. This variability in tokenization can further propagate semantic drift, where slight differences in phrasing result in divergent embeddings despite similar clinical meaning. These factors suggest that although BERT provides valuable semantic richness, its use must be balanced against concerns around efficiency, representational stability, and semantic fidelity in EHR settings.

Another methodological challenge lies in the joint optimization of binary classification (AUROC) and survival ranking (C-index) within a multitask framework. Although this design captures complementary dimensions of CVD risk, the 2 objectives are not always easily harmonized. In our results, C-index scores were consistently lower than AUROC, indicating difficulty in modeling TTE information with the same fidelity as event discrimination. In addition, evaluating a true 10-year TTE objective with censoring and aligning predictors with outcome timing typically yields a more conservative AUROC than shorter or time-gated tasks, which helps explain why our discrimination is lower than some reports. Future work may benefit from refined training strategies or alternative loss functions to better align these targets.

Model comparisons were conducted under a shared experimental framework established in our prior work [24], with consistent data extraction and preprocessing across all models. This enabled fair benchmarking but does not eliminate concerns about potential biases from repeated records or unobserved confounding. Given that the same CPRD dataset, variable and outcome definitions, cohort construction strategies, and censoring handling were applied, these models serve as appropriate comparators for evaluating the added value of MT-BERT. Within this like-for-like design, MT-BERT exceeded the conventional and ML baselines from our previous benchmark, with gains that were modest in absolute terms yet consistent across outcomes, and with clearer KM separation and better calibration. Independent replication in other settings will nonetheless be critical to validate generalizability.

The model also showed differential performance across outcomes. Prediction was most effective for composite CVD events, likely due to broader definitions and higher case counts [34]. In contrast, individual outcomes such as MI and vascular dementia were more difficult to model, reflecting data sparsity and noisier labels. MI is commonly harder because many events are precipitated by acute triggers that are not present in baseline features, which limits discriminability over a 10-year horizon. These findings highlight the practical value of composite endpoints in primary care, where follow-up is long and individual outcomes are rare. Future work will incorporate richer short-term and time-varying signals, for example, recent trajectories of vitals and laboratory results, medication changes, secondary-care encounters, and uncertainty-aware learning, to strengthen outcome-specific prediction.

Although generalizability was demonstrated between the development and spatial external validation sets, the model has not yet been evaluated across other health care systems or geographic regions. The London cohort provided important demographic diversity, especially in ethnicity and deprivation, but broader validation is required. Notably, performance declined in South Asian and socioeconomically deprived males, potentially due to lower case numbers and greater uncertainty. This highlights the need for targeted data enrichment and uncertainty-aware learning in underrepresented subgroups. Fairness-related issues also remain. While subgroup audits were conducted, mitigation strategies such as sample reweighting or recalibration were not applied, and systematic subgroup-specific auditing will be required before equitable clinical deployment can be ensured.

From an implementation perspective, the present work relied on DistilBERT, a relatively lightweight model pretrained on general rather than medical corpora. This choice reduced computational cost, allowing training within hours on high-performance computing environments and inference within seconds on standard CPUs, demonstrating feasibility for near real-time use in primary care dashboards, such as flagging high-risk patients for recall or displaying updated risk estimates during consultations. However, the absence of domain-specific pretraining remains a limitation, and larger biomedical language models trained on richer corpora are likely to achieve superior performance in the future. While deployment at scale is unlikely to pose major challenges, training requirements will ultimately depend on the size and complexity of the pretrained models used, and these aspects will need to be formally evaluated.

Interpretability also remains a key barrier to adoption. Deep learning models are often considered “black boxes,” in contrast to conventional statistical methods [6]. For structured variables, attribution methods such as SHapley Additive exPlanations (SHAP) can provide ranked contributions, enabling clinicians to see which risk factors (eg, blood pressure and diabetes status) were most influential. For clinical text, attention mechanisms can highlight salient tokens or phrases (eg, “chest pain” and “family history of stroke”) that received higher model weights, offering a complementary perspective. These cross-modal explanations require distinct visualization strategies, and we did not implement a systematic framework for presenting them to clinicians in this study. While such tools may improve explainability, the trade-off between predictive performance and interpretability must be carefully considered [35]. In contexts where textual data add limited value, simpler approaches such as RSF may provide comparable results with greater transparency. For long-term CVD prediction, robust risk stratification may ultimately be more clinically relevant than token-level explanations, yet future research should examine how to deliver outputs in formats that are both faithful to the model and usable in practice.

Importantly, this model is not intended to replace established risk tools such as QRISK4 [36], which have undergone extensive validation. Rather, it illustrates the potential of combining structured and unstructured EHR data within a multitask learning framework to support multioutcome prediction. Prospective validation in external health systems, integration into clinical care pathways, development of clinician-facing interpretability tools, and incorporation of fairness-aware strategies will be essential to translate these preliminary findings into real-world practice.

### Conclusion

This study presents a multitask deep learning model for 10-year CVD risk prediction using real-world EHR data, integrating structured variables and unstructured clinical text through an MT-BERT architecture. By jointly optimizing classification and survival objectives, the model achieves strong discrimination, robust calibration, and meaningful risk stratification over a 10-year follow-up. Performance remained consistent in a demographically diverse London subgroup, supporting its generalizability. Although improvements over previously tested ML-based models were modest, these findings provide preliminary evidence that combining representation learning and multiobjective optimization is a promising direction for risk prediction in primary care. However, subgroup differences across ethnicity and deprivation highlight persistent fairness concerns. Future work should focus on improving interpretability, validating in external populations, and evaluating clinical impact to support real-world implementation.

## Supplementary material

10.2196/76659 Multimedia Appendix 1 Supplementary tables providing detailed information on model features, disease definitions, software packages, and extended performance metrics of the multitask BERT model across cardiovascular disease outcomes by gender and dataset split.

10.2196/76659 Multimedia Appendix 2 Supplementary file containing phenotype definitions used in this study, including cardiovascular disease outcomes, comorbidities, and related risk factors, provided as individual CSV files.

10.2196/76659 Checklist 1 Transparent Reporting of a multivariable prediction model for Individual Prognosis Or Diagnosis checklist.

## Abbreviations

AI: artificial intelligence
AUROC: area under the receiver operating characteristic curve
BERT: Bidirectional Encoder Representations from Transformers
C-index: concordance index
CALIBER: Clinical research using Linked Bespoke studies and Electronic health Records
CALIBER: CArdiovascular disease research using LInked BEspoke studies and Electronic health Records
CHD: coronary heart disease
CPRD: Clinical Practice Research Datalink
CT: Systematized Nomenclature of Medicine – Clinical Terms
CVD: cardiovascular disease
DeepHit: Deep Neural Network for Competing Risks
DeepSurv: Deep Neural Network-based Cox Model
EHR: electronic health record
GBSA: Gradient Boosted Survival Analysis
GP: general practitioner
*ICD-10*: *International Classification of Diseases, Tenth Revision*
ISAC: Independent Scientific Advisory Committee
KM: Kaplan-Meier
ML: machine learning
MLP: multilayer perceptron
NICE: National Institute for Health and Care Excellence
NLP: natural language processing
RSF: Random Survival Forest
SBP: systolic blood pressure
SHAP: SHapley Additive exPlanations
TRIPOD: Transparent Reporting of a multivariable prediction model for Individual Prognosis Or Diagnosis
TTE: time-to-event
XGBS: Extreme Gradient Boosting Survival
XGBS: Extreme Gradient Boosted Survival Model

## References

[1] (2024). UK cardiovascular disease factsheet. British Heart Foundation.

[2] (2023). Number of people living with diabetes in the UK tops 5 million for the first time. Diabetes UK.

[3] (2023). Cardiovascular disease: risk assessment and reduction, including lipid modification report no: NG238. National Institute for Health and Care Excellence (NICE).

[4] Hippisley-Cox J, Coupland C, Brindle P (2017). Development and validation of QRISK3 risk prediction algorithms to estimate future risk of cardiovascular disease: prospective cohort study. BMJ.

[5] Virani SS, Newby LK, Arnold SV (2023). 2023 AHA/ACC/ACCP/ASPC/NLA/PCNA Guideline for the management of patients with chronic coronary disease: a report of the American Heart Association/American College of Cardiology Joint Committee on Clinical Practice Guidelines. Circulation.

[6] Liu T, Krentz A, Lu L, Curcin V (2025). Machine learning based prediction models for cardiovascular disease risk using electronic health records data: systematic review and meta-analysis. Eur Heart J Digit Health.

[7] Liu T, Krentz AJ, Huo Z, Ćurčin V (2025). Opportunities and challenges of cardiovascular disease risk prediction for primary prevention using machine learning and electronic health records: a systematic review. Rev Cardiovasc Med.

[8] Razieh C, Zaccardi F, Miksza J (2022). Differences in the risk of cardiovascular disease across ethnic groups: UK Biobank observational study. Nutr Metab Cardiovasc Dis.

[9] Eastwood SV, Mathur R, Sattar N, Smeeth L, Bhaskaran K, Chaturvedi N (2021). Ethnic differences in guideline-indicated statin initiation for people with type 2 diabetes in UK primary care, 2006-2019: a cohort study. PLoS Med.

[10] (2025). Socioeconomic inequalities in heart and circulatory diseases in england: an analysis. British Heart Foundation.

[11] Rajkomar A, Dean J, Kohane I (2019). Machine learning in medicine. N Engl J Med.

[12] Katzman JL, Shaham U, Cloninger A, Bates J, Jiang T, Kluger Y (2018). DeepSurv: personalized treatment recommender system using a Cox proportional hazards deep neural network. BMC Med Res Methodol.

[13] Shickel B, Tighe PJ, Bihorac A, Rashidi P (2018). Deep EHR: a survey of recent advances in deep learning techniques for electronic health record (EHR) analysis. IEEE J Biomed Health Inform.

[14] Devlin J, Chang MW, Lee K, Toutanova K (2019). BERT: pre-training of deep bidirectional transformers for language understanding. arXiv.

[15] (2022). CPRD Aurum January 2022.

[16] Collins GS, Moons KGM, Dhiman P (2024). TRIPOD+AI statement: updated guidance for reporting clinical prediction models that use regression or machine learning methods. BMJ.

[17] Riley RD, Snell KI, Ensor J (2019). Minimum sample size for developing a multivariable prediction model: PART II ‐ binary and time‐to‐event outcomes. Stat Med.

[18] Thayer DS, Mumtaz S, Elmessary MA (2024). Creating a next-generation phenotype library: the health data research UK Phenotype Library. JAMIA Open.

[19] Denaxas S, Gonzalez-Izquierdo A, Direk K (2019). UK phenomics platform for developing and validating electronic health record phenotypes: CALIBER. J Am Med Inform Assoc.

[20] Yan L (2019). QRISK3: 10-year cardiovascular disease risk calculator (QRISK3 2017). ClinRisk Ltd.

[21] Sanh V, Debut L, Chaumond J, Wolf T (2020). DistilBERT, a distilled version of BERT: smaller, faster, cheaper and lighter. arXiv.

[22] Kendall A, Gal Y, Cipolla R (2018). Multi-task learning using uncertainty to weigh losses for scene geometry and semantics. arXiv.

[23] Higgins JPT, Thompson SG, Deeks JJ, Altman DG (2003). Measuring inconsistency in meta-analyses. BMJ.

[24] Liu T, Krentz AJ, Lu L, Wang Y, Curcin V (2025). Benchmarking survival machine learning models for 10-year cardiovascular disease risk prediction using large-scale electronic health records. Digit Health.

[25] Davidson-Pilon C (2019). Lifelines: survival analysis in Python. J Open Source Softw.

[26] Lee C, Zame W, Yoon J, Van der Schaar M DeepHit: a deep learning approach to survival analysis with competing risks.

[27] Weng SF, Reps J, Kai J, Garibaldi JM, Qureshi N (2017). Can machine-learning improve cardiovascular risk prediction using routine clinical data?. PLoS ONE.

[28] Yang X, Chen A, PourNejatian N A large language model for electronic health records. npj Digit Med.

[29] Yang Z, Mitra A, Liu W, Berlowitz D, Yu H (2023). TransformEHR: transformer-based encoder-decoder generative model to enhance prediction of disease outcomes using electronic health records. Nat Commun.

[30] Alsentzer E, Murphy JR (2019). Publicly available clinical BERT embeddings. arXiv.

[31] Ruder S (2017). An overview of multi-task learning in deep neural networks. arXiv.

[32] Harutyunyan H, Khachatrian H, Kale DC, Ver Steeg G, Galstyan A (2019). Multitask learning and benchmarking with clinical time series data. Sci Data.

[33] Colacci M, Huang YQ, Postill G (2025). Sociodemographic bias in clinical machine learning models: a scoping review of algorithmic bias instances and mechanisms. J Clin Epidemiol.

[34] Rajkomar A, Oren E, Chen K (2018). Scalable and accurate deep learning with electronic health records. NPJ Digit Med.

[35] Lundberg S, Lee SI (2017). A unified approach to interpreting model predictions. arXiv.

[36] Hippisley-Cox J, Coupland CAC, Bafadhel M (2024). Development and validation of a new algorithm for improved cardiovascular risk prediction. Nat Med.

[37] Access to data. CPRD.

[38] TLiuBB/bert_paper. GitHub.

