# Supplementary material for: Estimating 10-Year Cardiovascular Disease Risk in Primary Prevention Using UK Electronic Health Records and a Hybrid Multitask BERT Model: Retrospective Cohort Study
Source: JMIR Med Inform. 2025 Nov 13;13:e76659. doi: 10.2196/76659 (PMC12620595; doi:10.2196/76659)
Supplement: Multimedia Appendix 1 [file medinform-v13-e76659-s001.docx]

**List of Supplementary Tables**

[Table S1 Comorbidities used in the models 2](#_Toc196786281)

[Table S2 Cardiovascular diseases outcomes used in the models 3](#_Toc196786282)

[Table S3 Key packages used in model development and evaluation 7](#_Toc196786283)

[Table S4 Performance metrics of the MT-BERT model across cardiovascular disease outcomes by gender (train, validate, test, and ‘spatial’ validate set) 8](#_Toc196786284)

| **ICD10** | **Description** | **Disease** | **Category** |
| --- | --- | --- | --- |
| **Atrial fibrillation** | | | |
| I48 | Atrial fibrillation and flutter | Atrial fibrillation | Diagnosis |
| **Erectile dysfunction** | | | |
| N48.4 | Impotence of organic origin | Erectile dysfunction | Diagnosis |
| F52.2 | Failure of genital response | Erectile dysfunction | Possible diagnosis |
| **HIV/AIDS** | | | |
| B20 | Human immunodeficiency virus [HIV] disease resulting in infectious and parasitic diseases | HIV | Diagnosis |
| B21 | Human immunodeficiency virus [HIV] disease resulting in malignant neoplasms | HIV | Diagnosis |
| B22 | Human immunodeficiency virus [HIV] disease resulting in other specified diseases | HIV | Diagnosis |
| B23 | Human immunodeficiency virus [HIV] disease resulting in other conditions | HIV | Diagnosis |
| B24 | Unspecified human immunodeficiency virus [HIV] disease | HIV | Diagnosis |
| F02.4 | Dementia in human immunodeficiency virus [HIV] disease | HIV | Diagnosis |
| R75 | Laboratory evidence of human immunodeficiency virus [HIV] | HIV | Diagnosis |
| Z21 | Asymptomatic human immunodeficiency virus [HIV] infection status | HIV | Diagnosis |
| **Migraine** | | | |
| G43 | Migraine | Migraine | Diagnosis |
| **Rheumatoid arthritis** | | | |
| J99.0 | Rheumatoid lung disease | Rheumatoid arthritis | Diagnosis |
| M05 | Seropositive rheumatoid arthritis | Rheumatoid arthritis | Diagnosis |
| M06 | Other rheumatoid arthritis | Rheumatoid arthritis | Diagnosis |
| **Lupus erythematosus** | | | |
| M32 | Systemic lupus erythematosus | Lupus erythematosus | Diagnosis |
| L93 | Lupus erythematosus | Lupus erythematosus | Diagnosis |

**Table S1** Comorbidities used in the models.

**Table S2** Cardiovascular diseases outcomes used in the models.

| **ICD10** | **Description** | **Disease** | **File** |
| --- | --- | --- | --- |
| **Coronary heart disease (angina and myocardial infarction)** | | | |
| I20 | Angina pectoris | Coronary heart disease | CHD |
| I200 | Unstable angina | Unstable angina | Angina unstable |
| I201 | Angina pectoris with documented spasm | Stable angina | Angina stable |
| I208 | Other forms of angina pectoris | Stable angina | Angina stable |
| I209 | Angina pectoris, unspecified | Stable angina | Angina stable |
| I21 | Acute myocardial infarction | Coronary heart disease  Myocardial infarction | CHD/MI |
| I22 | Subsequent myocardial infarction | Coronary heart disease  Myocardial infarction | CHD/MI |
| I23 | Certain current complications following acute myocardial infarction | Coronary heart disease  Myocardial infarction | CHD/MI |
| I24 | Other acute ischaemic heart diseases | Coronary heart disease | CHD |
| I241 | Dressler’s syndrome | Myocardial infarction | MI |
| I25 | Chronic ischaemic heart disease | Coronary heart disease | CHD |
| I250 | Atherosclerotic cardiovascular disease, so described | Coronary heart disease | CHD NOS Death |
| I251 | Atherosclerotic heart disease | Coronary heart disease | CHD NOS Death |
| I252 | Old myocardial infarction | Myocardial infarction | MI |
| I253 | Aneurysm of heart | Coronary heart disease | CHD NOS Death |
| I254 | Coronary artery aneurysm | Coronary heart disease | CHD NOS Death |
| I255 | Ischaemic cardiomyopathy | Coronary heart disease | CHD NOS Death |
| I256 | Silent myocardial ischaemia | Coronary heart disease | CHD NOS Death |
| I258 | Other forms of chronic ischaemic heart disease | Coronary heart disease | CHD NOS Death |
| I259 | Chronic ischaemic heart disease, unspecified | Coronary heart disease | CHD NOS Death |
| **Stroke and TIA** | | | |
| G45.0 | Vertebro-basilar artery syndrome | Transient ischaemic attack | TIA |
| G45.1 | Carotid artery syndrome (hemispheric) | Transient ischaemic attack | TIA |
| G45.2 | Multiple and bilateral precerebral artery syndromes | Transient ischaemic attack | TIA |
| G45.3 | Amaurosis fugax | Transient ischaemic attack | TIA |
| G45.4 | Transient global amnesia | Transient ischaemic attack | TIA |
| G45.8 | Other transient cerebral ischaemic attacks and related syndromes | Transient ischaemic attack | TIA |
| G45.9 | Transient cerebral ischaemic attack, unspecified | Transient ischaemic attack | TIA |
| G46.0 | Middle cerebral artery syndrome | Transient ischaemic attack | TIA |
| G46.1 | Anterior cerebral artery syndrome | Transient ischaemic attack | TIA |
| G46.2 | Posterior cerebral artery syndrome | Transient ischaemic attack | TIA |
| G46.3 | Brain stem stroke syndrome | Stroke | Stroke NOS |
| G46.4 | Cerebellar stroke syndrome | Stroke | Stroke NOS |
| G46.5 | Pure motor lacunar syndrome | Stroke | Stroke NOS |
| G46.6 | Pure sensory lacunar syndrome | Stroke | Stroke NOS |
| G46.7 | Other lacunar syndromes | Stroke | Stroke NOS |
| G46.8 | Other vascular syndromes of brain in cerebrovascular diseases | Stroke | Stroke NOS |
| I63.0 | Cerebral infarction due to thrombosis of precerebral arteries | Stroke | Stroke ischaemic |
| I63.1 | Cerebral infarction due to embolism of precerebral arteries | Stroke | Stroke ischaemic |
| I63.2 | Cerebral infarction due to unspecified occlusion or stenosis of precerebral arteries | Stroke | Stroke ischaemic |
| I63.3 | Cerebral infarction due to thrombosis of cerebral arteries | Stroke | Stroke ischaemic |
| I63.4 | Cerebral infarction due to embolism of cerebral arteries | Stroke | Stroke ischaemic |
| I63.5 | Cerebral infarction due to unspecified occlusion or stenosis of cerebral arteries | Stroke | Stroke ischaemic |
| I63.8 | Other cerebral infarction | Stroke | Stroke ischaemic |
| I63.9 | Cerebral infarction, unspecified | Stroke | Stroke ischaemic |
| I64 | Stroke, not specified as haemorrhage or infarction | Stroke | Stroke NOS |
| I65 | Occlusion and stenosis of precerebral arteries, not resulting in cerebral infarction | Transient ischaemic attack | TIA |
| I66 | Occlusion and stenosis of cerebral arteries, not resulting in cerebral infarction | Transient ischaemic attack | TIA |
| I69.3 | Sequelae of cerebral infarction | Stroke | Stroke ischaemic |
| I69.4 | Sequelae of stroke, not specified as haemorrhage or infarction | Stroke | Stroke NOS |
| **Other (hypertension, heart failure, AAA, and PAD)** | | | |
| I10 | Essential (primary) hypertension | Hypertension | Hypertension |
| I11 | Hypertensive heart disease | Hypertension | Hypertension |
| I11.0 | Hypertensive heart disease with (congestive) heart failure | Heart failure | HF |
| I12 | Hypertensive renal disease | Hypertension | Hypertension |
| I13 | Hypertensive heart and renal disease | Hypertension | Hypertension |
| I13.0 | Hypertensive heart and renal disease with (congestive) heart failure | Heart failure | HF |
| I13.2 | Hypertensive heart and renal disease with both (congestive) heart failure and renal failure | Heart failure | HF |
| I15 | Secondary hypertension | Hypertension | Hypertension |
| I50 | Heart failure | Heart failure | HF |
| I713 | Abdominal aortic aneurysm, ruptured | Abdominal aortic aneurysm | AAA |
| I714 | Abdominal aortic aneurysm, without mention of rupture | Abdominal aortic aneurysm | AAA |
| I715 | Thoracoabdominal aortic aneurysm, ruptured | Abdominal aortic aneurysm | AAA |
| I716 | Thoracoabdominal aortic aneurysm, without mention of rupture | Abdominal aortic aneurysm | AAA |
| I718 | Aortic aneurysm of unspecified site, ruptured | Abdominal aortic aneurysm | AAA |
| I719 | Aortic aneurysm of unspecified site, without mention of rupture | Abdominal aortic aneurysm | AAA |
| I731 | Thromboangiitis obliterans [Buerger] | Peripheral arterial disease | PAD |
| I738 | Other specified peripheral vascular diseases | Peripheral arterial disease | PAD |
| I739 | Peripheral vascular disease, unspecified | Peripheral arterial disease | PAD |
| I743 | Embolism and thrombosis of arteries of lower extremities | Peripheral arterial disease | PAD |
| I744 | Embolism and thrombosis of arteries of extremities, unspecified | Peripheral arterial disease | PAD |
| I745 | Embolism and thrombosis of iliac artery | Peripheral arterial disease | PAD |
| **Vascular dementia** | | | |
| F01 | Vascular dementia | Vascular dementia | VaD |
| F010 | Vascular dementia of acute onset | Vascular dementia | VaD |
| F011 | Multi-infarct dementia | Vascular dementia | VaD |
| F012 | Subcortical vascular dementia | Vascular dementia | VaD |
| F013 | Mixed cortical and subcortical vascular dementia | Vascular dementia | VaD |
| F018 | Other vascular dementia | Vascular dementia | VaD |
| F019 | Vascular dementia, unspecified | Vascular dementia | VaD |

**Table S3** Key packages used in model development and evaluation.

| **Package** | **Version** | **Specific Functions Used** | **Purpose** |
| --- | --- | --- | --- |
| Torch | 2.3.1.post100 | nn.Module, nn.functional.cross_entropy, exp, cumsum, log, nn.functional.softplus, optim.AdamW, TensorDataset, DataLoader, clip_grad_norm_, nn.MultiheadAttention, nn.LayerNorm | Model architecture, loss computation (Focal and Cox losses), optimization, and training utilities. |
| transformers | 4.45.2 | DistilBertTokenizer, DistilBertModel | Tokenization and pre-trained BERT model encoding. |
| scikit-learn | 1.5.2 | train_test_split, MinMaxScaler, compute_class_weight, roc_auc_score, precision_score, recall_score, f1_score, precision_recall_curve, auc, accuracy_score | Data splitting, feature scaling, class balancing, and evaluation metrics computation. |
| optuna | 4.1.0 | create_study, TrialState | Hyperparameter tuning through Bayesian optimization. |
| lifelines | 0.30.0 | concordance_index | Computation of C-index for survival analysis performance evaluation. |
| pandas | 2.2.3 | read_csv, DataFrame operations | Data loading and preprocessing. |
| numpy | 1.26.4 | array, percentile, random seed setting | Numerical operations and random seed initialization. |

**Table S4** Performance metrics of the MT-BERT model across cardiovascular disease outcomes by gender (train, validate, test, and ‘spatial’ validate set).

| **Label - Split** | **AUROC**  **(95% CI)** | **C-index**  **(95% CI)** | **Brier score** | **Accuracy** | **Specificity** | **Recall** | **Baseline threshold (%)** |
| --- | --- | --- | --- | --- | --- | --- | --- |
| **Men** | | | | | | | |
| **CVD (QRISK) - train** | 0.7309  (0.7279, 0.734) | 0.7039  (0.7019, 0.7054) | 0.1192 | 0.8489 | 0.895 | 0.3325 | 0.38 |
| **CVD (QRISK) - validate** | 0.7249  (0.7186, 0.732) | 0.7014  (0.6987, 0.7042) | 0.1194 | 0.7636 | 0.7855 | 0.5183 | 0.35 |
| **CVD (QRISK) - test** | 0.7227  (0.7172, 0.732) | 0.699  (0.6961, 0.7017) | 0.1193 | 0.7331 | 0.7496 | 0.5475 | 0.34 |
| **CVD (QRISK) - external validate** | 0.736  (0.726, 0.7416) | 0.7073  (0.7044, 0.7103) | 0.1142 | 0.8228 | 0.8516 | 0.4301 | 0.37 |
| **CVD (Composite) - train** | 0.7415  (0.7392, 0.7458) | 0.7138  (0.7122, 0.7149) | 0.1299 | 0.82 | 0.8699 | 0.4106 | 0.4 |
| **CVD (Composite) - validate** | 0.7454  (0.7403, 0.7523) | 0.7172  (0.7131, 0.7206) | 0.1296 | 0.7588 | 0.7821 | 0.5679 | 0.36 |
| **CVD (Composite) - test** | 0.7438  (0.7384, 0.7487) | 0.7125  (0.7091, 0.7156) | 0.1298 | 0.8231 | 0.874 | 0.4051 | 0.4 |
| **CVD (Composite) - external validate** | 0.7456  (0.7387, 0.7506) | 0.7041  (0.7019, 0.7083) | 0.1226 | 0.8284 | 0.869 | 0.4196 | 0.39 |
| **CHD - train** | 0.7206  (0.7161, 0.7241) | 0.704  (0.7021, 0.7058) | 0.1072 | 0.8339 | 0.8569 | 0.3765 | 0.35 |
| **CHD - validate** | 0.7267  (0.7192, 0.7358) | 0.7044  (0.7015, 0.7083) | 0.1072 | 0.9129 | 0.9483 | 0.2098 | 0.36 |
| **CHD - test** | 0.7318  (0.7258, 0.739) | 0.7069  (0.7037, 0.7094) | 0.1072 | 0.782 | 0.7953 | 0.518 | 0.34 |
| **CHD - external validate** | 0.7282  (0.7186, 0.7357) | 0.7102  (0.7071, 0.7125) | 0.1016 | 0.8909 | 0.9128 | 0.3105 | 0.35 |
| **Stroke - train** | 0.735  (0.7249, 0.7475) | 0.6864  (0.6845, 0.6878) | 0.0652 | 0.6733 | 0.6733 | 0.6644 | 0.33 |
| **Stroke - validate** | 0.7061  (0.6859, 0.7372) | 0.6821  (0.6787, 0.6859) | 0.0652 | 0.6769 | 0.6772 | 0.6143 | 0.33 |
| **Stroke - test** | 0.7382  (0.7021, 0.7643) | 0.6882  (0.684, 0.6918) | 0.065 | 0.6763 | 0.6764 | 0.6562 | 0.33 |
| **Stroke - external validate** | 0.7407  (0.7211, 0.7659) | 0.6884  (0.6858, 0.6914) | 0.0661 | 0.6711 | 0.6711 | 0.6703 | 0.33 |
| **MI - train** | 0.6917  (0.685, 0.6952) | 0.6643  (0.6624, 0.6661) | 0.1326 | 0.1244 | 0.1022 | 0.9597 | 0.35 |
| **MI - validate** | 0.6874  (0.6707, 0.6986) | 0.6626  (0.6593, 0.6654) | 0.1326 | 0.2223 | 0.2035 | 0.926 | 0.35 |
| **MI - test** | 0.6793  (0.6699, 0.6938) | 0.6669  (0.6627, 0.6739) | 0.1326 | 0.1246 | 0.1024 | 0.958 | 0.35 |
| **MI - external validate** | 0.6919  (0.6745, 0.7003) | 0.6839  (0.6815, 0.6877) | 0.1304 | 0.2181 | 0.203 | 0.9117 | 0.35 |
| **Angina - train** | 0.7182  (0.7145, 0.7226) | 0.6991  (0.6976, 0.701) | 0.1035 | 0.9262 | 0.9556 | 0.1787 | 0.35 |
| **Angina - validate** | 0.7187  (0.71, 0.7297) | 0.6992  (0.6949, 0.7046) | 0.1036 | 0.9257 | 0.9553 | 0.1712 | 0.35 |
| **Angina - test** | 0.7126  (0.7021, 0.7225) | 0.701  (0.6952, 0.7061) | 0.1035 | 0.7648 | 0.7746 | 0.5165 | 0.33 |
| **Angina - external validate** | 0.7196  (0.7095, 0.7287) | 0.6742  (0.6704, 0.6768) | 0.101 | 0.8631 | 0.8783 | 0.3678 | 0.34 |
| **Women** | | | | | | | |
| **CVD (QRISK) - train** | 0.7729  (0.7691, 0.7769) | 0.7526  (0.7506, 0.7537) | 0.0768 | 0.9081 | 0.9391 | 0.3084 | 0.31 |
| **CVD (QRISK) - validate** | 0.7753  (0.7672, 0.785) | 0.7494  (0.7454, 0.7529) | 0.0768 | 0.9039 | 0.9336 | 0.3291 | 0.31 |
| **CVD (QRISK) - test** | 0.7673  (0.7598, 0.7774) | 0.7521  (0.7485, 0.7567) | 0.077 | 0.9137 | 0.9456 | 0.295 | 0.31 |
| **CVD (QRISK) - external validate** | 0.7926  (0.7848, 0.7996) | 0.7475  (0.745, 0.7511) | 0.0679 | 0.9249 | 0.9474 | 0.317 | 0.3 |
| **CVD (Composite) - train** | 0.794  (0.7888, 0.8026) | 0.735  (0.7324, 0.7385) | 0.0903 | 0.8648 | 0.8919 | 0.4842 | 0.34 |
| **CVD (Composite) - validate** | 0.7829  (0.7677, 0.7975) | 0.7351  (0.7273, 0.7431) | 0.0909 | 0.8713 | 0.9015 | 0.4464 | 0.34 |
| **CVD (Composite) - test** | 0.7819  (0.7681, 0.7964) | 0.7322  (0.7251, 0.7416) | 0.0905 | 0.8608 | 0.8873 | 0.4874 | 0.34 |
| **CVD (Composite) - external validate** | 0.81  (0.803, 0.8152) | 0.7291  (0.7267, 0.7325) | 0.079 | 0.9306 | 0.9629 | 0.3124 | 0.34 |
| **CHD - train** | 0.7554  (0.7436, 0.7653) | 0.7316  (0.7289, 0.735) | 0.0592 | 0.9481 | 0.9672 | 0.1618 | 0.29 |
| **CHD - validate** | 0.7404  (0.7198, 0.7606) | 0.7364  (0.7311, 0.7442) | 0.0594 | 0.892 | 0.9057 | 0.3333 | 0.28 |
| **CHD - test** | 0.769  (0.7504, 0.7848) | 0.732  (0.7246, 0.742) | 0.0594 | 0.8724 | 0.8833 | 0.4266 | 0.28 |
| **CHD - external validate** | 0.7736  (0.7612, 0.781) | 0.7183  (0.7165, 0.7218) | 0.0514 | 0.9541 | 0.9661 | 0.1771 | 0.28 |
| **Stroke - train** | 0.7528  (0.7192, 0.7914) | 0.7379  (0.7344, 0.7419) | 0.0182 | 0.9368 | 0.9391 | 0.2308 | 0.23 |
| **Stroke - validate** | 0.7445  (0.6998, 0.7982) | 0.735  (0.7271, 0.7426) | 0.0182 | 0.8382 | 0.8394 | 0.4615 | 0.19 |
| **Stroke - test** | 0.7688  (0.7172, 0.8528) | 0.737  (0.7321, 0.7447) | 0.0182 | 0.9342 | 0.9364 | 0.2564 | 0.23 |
| **Stroke - external validate** | 0.7528  (0.7356, 0.7774) | 0.7502  (0.7468, 0.7528) | 0.0172 | 0.9077 | 0.9096 | 0.3552 | 0.22 |
| **MI - train** | 0.7134  (0.696, 0.7313) | 0.6886  (0.685, 0.6931) | 0.0779 | 0.3081 | 0.3023 | 0.9373 | 0.27 |
| **MI - validate** | 0.6926  (0.6436, 0.7205) | 0.6962  (0.687, 0.7059) | 0.0779 | 0.2086 | 0.2016 | 0.9725 | 0.27 |
| **MI - test** | 0.7168  (0.6803, 0.7606) | 0.6873  (0.682, 0.6959) | 0.078 | 0.4079 | 0.4033 | 0.9 | 0.27 |
| **MI - external validate** | 0.7395  (0.7254, 0.7537) | 0.7114  (0.7081, 0.7148) | 0.077 | 0.4058 | 0.4022 | 0.8754 | 0.27 |
| **Angina - train** | 0.7487  (0.7418, 0.753) | 0.721  (0.72, 0.7231) | 0.0701 | 0.9551 | 0.9712 | 0.1456 | 0.3 |
| **Angina - validate** | 0.7518  (0.7381, 0.7662) | 0.7225  (0.7188, 0.7254) | 0.0701 | 0.8921 | 0.9034 | 0.3276 | 0.29 |
| **Angina - test** | 0.7409  (0.7305, 0.7538) | 0.7208  (0.7175, 0.7241) | 0.0701 | 0.8776 | 0.8874 | 0.3873 | 0.29 |
| **Angina - external validate** | 0.7749  (0.7622, 0.7823) | 0.7038  (0.7007, 0.708) | 0.0651 | 0.9697 | 0.982 | 0.1143 | 0.3 |
